# Supplementary material for: Taking Up and Terminating Leisure-Time Physical Activity over the Life Course: The Role of Life Events in the Familial and Occupational Life Domains
Source: Int J Environ Res Public Health. 2021 Sep 17;18(18):9809. doi: 10.3390/ijerph18189809 (PMC8468990; doi:10.3390/ijerph18189809)
Supplement: Supplementary file 1 [file ijerph-18-09809-s001.zip › ijerph-1382187-supplementary.pdf]

Supplementary Table S1: Values of the Krippendorff's alpha for the test–retest–reliability (*n* = 29).

Variable	Scale	Krippendorff's alpha	
		Point estimate	*Bootstrap 95%–CI*[1]
Taking up & terminating LTPA in life course			
First time taking up LTPA in life course (age)	ratio	.81	.72 – .89
Interruption(s) of LTPA in life course (yes/no)	nominal	.90	.70 – 1.00
Number of interruptions of LTPA in life course	ratio	.88	.67 – 1.00
Timing of interruptions of LTPA in life course (age)	ratio	.98	.94 – 1.00
Life events from the familial life domain (last 15 years)			
Number of past relationships	ratio	.90	.77 – 1.00
Timing of relationships (start & end; age)	ratio	.99	.99 – 1.00
Number of children	ratio	1.00	–
Birth year of children	interval	1.00	–
Life events from the occupational life domain (last 15 years)			
Number of the vocational trainings	ratio	.92	.77 – 1.00
Timing of vocational trainings (start & end; age)	ratio	.99	.99 – 1.00
Number of jobs	ratio	.96	.91 – 1.00
Timing of jobs (start & end; age)	ratio	.99	.96 – 1.00
Timing of retirement (age)	ratio	.98	.93 – 1.00

Note: Krippendorff's alpha is for a nominal scale similar to Scott's Pi and for an interval scale similar to Pearson et al.'s intraclass–correlation coefficient (see Hayes & Krippendorff, 2007 for further information).

[1]As suggested (Hayes & Krippendorff, 2007), 10'000 bootstrap sampling distributions were done (CI = confidence interval).

Supplementary Table S2: Multilevel discrete–time event–history analysis for **starting a relationship on taking up LTPA**. The model presented is calculated without interaction effects for the life event with gender and/or age groups, because this does not improve model fit.

Fixed effects	logit	*SE*	*z* value	*P*
Intercept	–1.19	0.53	–2.26	.02
Starting a relationship (12–72 years)	0.64	0.51	1.25	.21
Control variables				
Sex (0 = male; 1 = female)	0.16	0.32	.51	.61
Level of education (1–5)	–0.09	0.13	–0.65	.52
Previous inactivity duration (1–15)	0.19	0.08	2.33	.02
Quotient active years	6.43	1.68	3.82	< .001
age group (ref. = 1; <30 y.)				
age group 2 (30–44 y.)	–0.71	0.52	–1.37	.17
age group 3 (45–59 y.)	–2.31	0.61	–3.81	< .001
age group 4 (60–72 y.)	–2.20	0.58	–3.77	< .001
Time (ref. = 8)				
Time 1	–1.41	0.80	–1.76	.08
Time 2	–0.29	0.66	–0.43	.67
Time 3	–0.73	0.75	–0.98	.33
Time 4	–0.25	0.67	–0.38	.71
Time 5	–0.41	0.70	–0.58	.56
Time 6	0.24	0.64	0.38	.70
Time 7	–1.01	0.76	–1.33	.18
Time 9	0.89	0.57	1.57	.12
Time 10	–1.58	0.90	–1.76	.08
Time 11	–0.03	0.63	–0.05	.96
Time 12	1.04	0.58	1.80	.07
Time 13	0.81	0.64	1.26	.21
Time 14	0.90	0.69	1.29	.20
Time 15	0.75	0.70	1.07	.29
Time 16	2.37	0.76	3.10	.002
Random effects	*Variance*	*SD*		
Person	0.93	0.96		

Note: *N* = 804 observations, *n* = 152 persons; SE = standard error, SD = standard deviation.

Supplementary Table S3: Multilevel discrete–time event–history analysis for **ending a relationship on taking up LTPA**. The model presented is calculated without interaction effects for the life event with gender and/or age groups, because this does not improve model fit.

Fixed effects	logit	*SE*	*z* value	*P*
Intercept	–1.81	0.40	–4.50	< .001
Ending a relationship (12–72 years)	1.31	0.44	3.00	.003
Control variables				
Sex (0 = male; 1 = female)	–0.14	0.17	–0.81	.42
Level of education (1–5)	0.11	0.06	1.73	.08
Previous inactivity duration (1–15)	–0.04	0.02	–1.97	.05
Quotient active years	1.85	0.43	4.33	< .001
age group (ref. = 1; <30 y.)				
age group 2 (30–44 y.)	0.31	0.27	1.15	.25
age group 3 (45–59 y.)	–0.28	0.28	–1.00	.25
age group 4 (60–72 y.)	–0.47	0.33	–1.42	.16
Time (ref. = 8)				
Time 1	–0.55	0.42	–1.32	.19
Time 2	–0.19	0.39	–0.48	.63
Time 3	–0.24	0.41	–0.60	.55
Time 4	–0.76	0.44	–1.72	.09
Time 5	–0.03	0.40	–0.08	.93
Time 6	–0.26	0.42	–0.62	.54
Time 7	–0.32	0.42	–0.77	.44
Time 9	0.07	0.41	0.17	.86
Time 10	0.31	0.41	0.75	.45
Time 11	0.64	0.40	1.62	.11
Time 12	0.08	0.43	0.19	.85
Time 13	0.45	0.42	1.06	.29
Time 14	0.32	0.43	0.73	.46
Time 15	1.30	0.42	3.07	.002
Time 16	1.81	0.50	3.66	< .001
Random effects	*Variance*	*SD*		
Person	0.05	0.23		

Note: *N* = 1586 observations, *n* = 264 persons; SE = standard error, SD = standard deviation.

Supplementary Table S4: Multilevel discrete–time event–history analysis for **becoming a parent on taking up LTPA**. The model presented is calculated without interaction effects for the life event with gender and/or age groups, because this does not improve model fit.

Fixed effects	logit	*SE*	*z* value	*P*
Intercept	–1.41	0.34	–4.14	< .001
Becoming a parent (15–54 years)	–1.06	0.45	2.34	.02
Control variables				
Sex (0 = male; 1 = female)	–0.21	0.17	–1.23	.22
Level of education (1–5)	0.06	0.07	0.84	.40
Previous inactivity duration (1–15)	0.01	0.02	0.07	.95
Quotient active years	2.07	0.47	4.36	< .001
age group (ref. = 1; <30 y.)				
age group 2 (30–44 y.)	0.17	0.20	0.85	.40
age group 3 (45–54 y.)	–0.57	0.23	–2.54	.01
Time (ref. = 8)				
Time 1	–0.53	0.41	–1.31	.19
Time 2	–0.18	0.39	–0.46	.65
Time 3	–0.42	0.42	–1.01	.31
Time 4	–0.55	0.42	–1.29	.20
Time 5	0.26	0.38	0.68	.49
Time 6	–0.15	0.42	–0.35	.73
Time 7	–0.46	0.45	–1.02	.31
Time 9	0.56	0.39	1.42	.16
Time 10	–0.14	0.45	–0.31	.75
Time 11	0.75	0.41	1.84	.07
Time 12	0.59	0..42	1.41	.16
Time 13	0.70	0.43	1.63	.10
Time 14	0.53	0.45	1.19	.23
Time 15	1.56	0.46	3.41	< .001
Time 16	3.37	0.74	4.55	< .001
Random effects	*Variance*	*SD*		
Person	0.08	0.29		

Note: N = 1391 observations, n = 280 persons; SE = standard error, SD = standard deviation.

Supplementary Table S5: Multilevel discrete–time event–history analysis for **starting vocational training on taking up LTPA.** The model presented is calculated without interaction effects for the life event with gender and/or age groups, because this does not improve model fit.

Fixed effects	logit	*SE*	*z* value	*P*
Intercept	4.77	1.83	2.60	.01
Starting vocational training (15–44 years)	–0.05	0.46	–0.11	.91
Control variables				
Sex (0 = male; 1 = female)	–0.28	0.23	–1.20	.23
Level of education (1–5)	0.02	0.09	0.22	.82
Previous inactivity duration (1–15)	<0.01	0.03	0.05	.96
Quotient active years	2.34	0.61	3.84	< .001
age group (ref. = 1; <30 y.)				
age group 2 (30–44 y.)	0.16	0.24	0.67	.50
Time (ref. = 8)				
Time 1	–0.33	0.57	–0.58	.56
Time 2	0.18	0.55	0.33	.74
Time 3	–0.33	0.60	–0.56	.58
Time 4	–0.47	0.61	–0.77	.44
Time 5	0.66	0.55	1.20	.23
Time 6	0.45	0.58	0.78	.44
Time 7	0.07	0.62	0.11	.91
Time 9	0.92	0.58	1.59	.11
Time 10	–0.44	0.71	–0.61	.54
Time 11	0.73	0.60	1.22	.22
Time 12	0.94	0.61	1.54	.12
Time 13	0.96	0.64	1.48	.14
Time 14	1.50	0.64	2.33	.02
Time 15	1.52	0.67	2.27	.02
Time 16	3.55	0.95	3.74	< .001
Random effects	*Variance*	*SD*		
Person	0.11	0.33		

Note: *N* = 759 observations, *n* = 183 persons; SE = standard error, SD = standard deviation.

Supplementary Table S6: Multilevel discrete–time event–history analysis for **starting a job on taking up LTPA**. The model presented is calculated without interaction effects for the life event with gender and/or age groups, because this does not improve model fit.

Fixed effects	logit	*SE*	*z* value	*P*
Intercept	4.44	1.18	3.77	< .001
Starting a job (15–70 years)	0.12	0.25	0.48	.63
Control variables				
Sex (0 = male; 1 = female)	–0.13	0.15	–0.86	.39
Level of education (1–5)	0.05	0.06	0.90	.37
Previous inactivity duration (1–15)	–0.02	0.02	–0.93	.35
Quotient active years	2.11	0.40	5.35	< .001
age group (ref. = 1; <30 y.)				
age group 2 (30–44 y.)	0.07	0.19	0.34	.73
age group 3 (45–59 y.)	–0.61	0.20	–2.99	.003
age group 4 (60–70 y.)	–0.78	0.28	–2.79	.005
Time (ref. = 8)				
Time 1	–0.67	0.38	–1.77	.08
Time 2	–0.35	0.35	–1.00	.32
Time 3	–0.27	0.36	–0.75	.45
Time 4	–0.61	0.38	–1.62	.11
Time 5	–0.06	0.35	–0.19	.85
Time 6	–0.10	0.36	–0.29	.77
Time 7	–0.50	0.39	–1.28	.20
Time 9	0.58	0.34	1.71	.09
Time 10	0.02	0.38	0.06	.95
Time 11	0.52	0.36	1.47	.14
Time 12	0.52	0.36	1.45	.15
Time 13	0.49	0.37	1.33	.18
Time 14	0.34	0.39	0.88	.38
Time 15	1.28	0.37	3.41	< .001
Time 16	2.19	0.45	4.90	< .001
Random effects	*Variance*	*SD*		
Person	0.06	0.24		

Note: *N* = 2025 observations, *n* = 342 persons; SE = standard error, SD = standard deviation.

Supplementary Table S7: Multilevel discrete–time event–history analysis for **ending a job on taking up LTPA**. The model presented is calculated without interaction effects for the life event with gender and/or age groups, because this does not improve model fit.

Fixed effects	logit	*SE*	*z* value	*P*
Intercept	4.97	1.20	4.15	< .001
Ending a job (15–70 years)	0.17	0.27	0.61	.54
Control variables				
Sex (0 = male; 1 = female)	–0.12	0.15	–0.79	.43
Level of education (1–5)	0.05	0.06	0.87	.38
Previous inactivity duration (1–15)	–0.01	0.02	–0.66	.51
Quotient active years	2.28	0.40	5.67	< .001
age group (ref. = 1; <30 y.)				
age group 2 (30–44 y.)	0.05	0.19	0.24	.81
age group 3 (45–59 y.)	–0.65	0.29	–3.21	.001
age group 4 (60–70 y.)	–1.08	0.30	–3.57	< .001
Time (ref. = 8)				
Time 1	–0.70	0.38	–1.85	.06
Time 2	–0.39	0.36	–1.09	.28
Time 3	–0.36	0.36	–1.00	.32
Time 4	–0.64	0.38	–1.68	.09
Time 5	–0.08	0.35	–0.23	.82
Time 6	–0.18	0.37	–0.48	.63
Time 7	–0.57	0.40	–1.45	.15
Time 9	0.43	0.35	1.22	.22
Time 10	–0.01	0.38	–0.03	.98
Time 11	0.50	0.36	1.40	.16
Time 12	0.39	0.37	1.05	.29
Time 13	0.49	0.37	1.30	.19
Time 14	0.34	0.39	0.85	.39
Time 15	1.30	0.38	3.43	< .001
Time 16	2.16	0.45	4.78	< .001
Random effects	*Variance*	*SD*		
Person	0.66	0.81		

Note: *N* = 1995 observations, *n* = 342 persons; SE = standard error, SD = standard deviation.

Supplementary Table S8: Multilevel discrete–time event–history analysis for **retirement on taking up LTPA**. The model presented is calculated without interaction effects for the life event with gender and/or age groups, because this does not improve model fit.

Fixed effects	logit	*SE*	*z* value	*P*
Intercept	2.21	1.90	1.16	.25
Retirement (50–72 years)	1.49	0.44	3.39	< .001
Control variables				
Sex (0 = male; 1 = female)	–0.02	0.25	–0.09	.93
Level of education (1–5)	0.12	0.10	1.32	.19
Previous inactivity duration (1–15)	–0.08	0.04	–2.22	.03
Quotient active years	1.42	0.70	2.04	.04
age group (ref. = 1; 50–59 y.)				
age group 4 (60–72 y.)	–0.17	0.29	–0.58	.60
Time (ref. = 8)				
Time 1	–0.91	0.71	–1.27	.20
Time 2	–0.93	0.71	–1.31	.19
Time 3	–0.17	0.54	–0.31	.76
Time 4	–0.22	0.55	–0.39	.70
Time 5	–1.57	0.82	–1.92	.05
Time 6	–0.71	0.58	–1.23	.22
Time 7	–1.41	0.65	–2.18	.03
Time 9	0.27	0.50	0.54	.59
Time 10	0.36	0.52	0.70	.48
Time 11	0.01	0.55	0.02	.99
Time 12	–0.06	0.55	–0.11	.91
Time 13	–0.31	0.60	–0.52	.60
Time 14	–0.30	0.62	–0.48	.63
Time 15	0.49	0.54	0.90	.37
Time 16	1.59	0.60	2.63	.009
Random effects	*Variance*	*SD*		
Person	0.12	0.36		

Note: *N* = 935 observations, *n* = 165 persons; SE = standard error, SD = standard deviation.

Supplementary Table S9: Multilevel discrete–time event–history analysis for **simultaneously occurring life events on taking up LTPA**. The model presented due to the best fit is calculated with the interaction effects for simultaneously occurred life events × age group, whereas the interaction with sex does not improve model fit.

Fixed effects	logit	*SE*	*z* value	*P*
Intercept	5.57	1.16	4.79	< .001
Simul. occurring life events (15–70 years)				
Simul. occurring life events × age group (ref. = 1; <30 y.)	–0.15	0.19	–0.79	.43
age group 2 (30–44 y.)	0.21	0.26	0.81	.42
age group 3 (45–59 y.)	0.67	0.29	2.29	.02
age group 4 (60–70 y.)	1.20	0.42	2.88	.004
changing reference category[1]				
Simul. occurreing life events × age group (ref. = 2; 30–44 y.)	0.06	0.18	0.33	.74
Simul. occurring life events × age group (ref. = 3; 4 5–59 y.)	0.52	0.22	2.33	.02
Simul. occurring life events × age group (ref. = 4; 60–70 y.)	1.05	0.37	2.83	.004
Control variables				
Sex (0 = male; 1 = female)	–0.13	0.14	–0.91	.36
Level of education (1–5)	0.04	0.05	0.71	.48
Previous inactivity duration (1–15)	0.01	0.02	–0.34	.74
Quotient active years	2.44	0.39	6.23	< .001
age group (ref. = 1; <30 y.)				
age group 2 (30–44 y.)	–0.04	0.22	–0.19	.85
age group 3 (45–59 y.)	–0.82	0.23	–3.64	< .001
age group 4 (60–70 y.)	–1.18	0.28	–4.22	< .001
Time (ref. = 8)				
Time 1	–0.84	0.37	–2.24	.03
Time 2	–0.38	0.34	–1.11	.27
Time 3	–0.36	0.35	–1.02	.31
Time 4	–0.70	0.37	–1.89	.06
Time 5	–0.23	0.35	–0.65	.52
Time 6	–0.26	0.35	–0.72	.47
Time 7	–0.51	0.37	–1.39	.16
Time 9	0.41	0.33	1.24	.22
Time 10	–0.09	0.37	–0.24	.81
Time 11	0.41	0.34	1.21	.23
Time 12	0.37	0.35	1.07	.29

	Time 13	0.44	0.35	1.23	.22
	Time 14	0.25	0.37	0.69	.49
	Time 15	1.10	0.36	3.07	.002
	Time 16	2.06	0.41	4.99	< .001
Random effects		*Variance*	*SD*		
Person		0.67	0.82		

Note: N = 2149 observations, n = 341 persons; SE = standard error, SD = standard deviation.

[1]To compare persons with and without experiencing a life event within the age group, the reference category was changed to this age group (Jaccard, 2001). The model and its values stays the same.

Supplementary Table S10: Multilevel discrete–time event–history analysis for **starting a relationship on terminating LTPA**. The model presented is calculated without interaction effects for the life event with gender and/or age groups, because this does not improve model fit.

Fixed effects	logit	*SE*	*z* value	*P*
Intercept	–6.50	1.12	–5.79	< .001
Starting a relationship (12–72 years)	0.64	0.46	1.39	.16
Control variables				
Sex (0 = male; 1 = female)	0.16	0.31	0.50	.62
Level of education (1–5)	–0.22	0.13	–1.68	.09
Previous activity duration (1–15)	0.07	0.07	1.07	.29
Quotient active years	–3.58	1.33	–2.69	.007
age group (ref. = 1; <30 y.)				
age group 2 (30–44 y.)	–0.52	0.46	–1.12	.26
age group 3 (45–59 y.)	–1.43	0.51	–2.81	.005
age group 4 (60–72 y.)	–1.75	0.56	–3.15	.001
Time (ref. = 8)				
Time 1	1.37	1.17	1.17	.24
Time 2	1.32	1.20	1.10	.27
Time 3	0.84	1.26	0.67	.50
Time 4	1.81	1.13	1.59	.11
Time 5	0.11	1.45	0.08	.94
Time 6	1.93	1.12	1.73	.08
Time 7	1.94	1.12	1.74	.08
Time 9	2.11	1.09	1.94	.05
Time 10	1.64	1.12	1.47	.14
Time 11	2.06	1.09	1.88	.06
Time 12	2.11	1.09	1.93	.05
Time 13	1.88	1.10	1.71	.09
Time 14	2.22	1.09	2.03	.04
Time 15	1.88	1.11	1.69	.09
Random effects	*Variance*	*SD*		
Person	2.47	1.57		

Note: *N* = 5427 observations, *n* = 696 persons; SE = standard error, SD = standard deviation; Time 16 had to be removed from the calculation because there are no terminations there.

Supplementary Table S11: Multilevel discrete–time event–history analysis for **ending a relationship on terminating LTPA**. The model presented due to the best fit is calculated with the interaction effects for ending a relationship × sex, whereas the interaction with age group does not improve model fit.

Fixed effects	logit	*SE*	*z* value	*P*
Intercept	–4.60	0.46	–9.97	< .001
Ending a relationship (12–72 years)				
Ending a Relationship × sex (ref. = men)	1.71	0.57		.003
sex (women)	–2.52	1.17		.03
changing reference category[1]				
Ending a Relationship × sex (ref. = women)	–0.82	1.02	–0.80	.43
Control variables				
Sex (0 = male; 1 = female)	0.55	0.19	2.88	.003
Level of education (1–5)	0.07	0.07	1.02	.31
Previous activity duration (1–15)	–0.10	0.03	–3.90	< .001
Quotient active years	–0.18	0.46	–0.39	.69
age group (ref. = 1; <30 y.)				
age group 2 (30–44 y.)	–0.66	0.29	–2.29	.02
age group 3 (45–59 y.)	–0.92	0.28	–3.24	.001
age group 4 (60–72 y.)	–1.66	0.35	–4.71	< .001
Time (ref. = 8)				
Time 1	0.28	0.44	0.64	.52
Time 2	–0.02	0.47	–0.05	.96
Time 3	0.41	0.43	0.95	.34
Time 4	–0.89	0.61	–1.48	.14
Time 5	0.14	0.46	0.31	.76
Time 6	0.37	0.43	0.84	.40
Time 7	0.32	0.44	0.72	.47
Time 9	–0.57	0.56	–1.01	.31
Time 10	0.12	0.47	0.26	.80
Time 11	0.69	0.42	1.64	.10
Time 12	0.22	0.46	0.48	.63
Time 13	0.31	0.45	0.70	.49
Time 14	0.31	0.45	0.69	.49
Time 15	0.06	0.48	0.13	.90
Random effects	*Variance*	*SD*		
Person	0.44	0.66		

Note: *N* = 17741 observations, *n* = 1423 persons; SE = standard error, SD = standard deviation; Time 16 had to be removed from the calculation because there are no terminations there.

[1]To compare persons with and without experiencing a life event within the age group, the reference category was changed to this age group (Jaccard, 2001). The model and its values stays the same.

Supplementary Table S12: Multilevel discrete–time event–history analysis for **becoming a parent on terminating LTPA**. The model presented due to the best fit is calculated with the interaction effects for becoming a parent × age group and becoming a parent × sex.

Fixed effects	logit	*SE*	*z* value	*P*
Intercept	–4.48	0.41	–10.89	< .001
Becoming a parent (15–54 years)				
Becoming a parent × age group (ref. = 1; <30 y.); Becoming a parent × sex (ref. = men)	–1.89	1.25		.13
age group 2 (30–44 y.)	1.57	0.72		.03
age group 3 (45–54 y.)	2.50	1.34		.06
sex (women)	3.15	1.09		.004
changing reference category[1]				
Becoming a parent × age group (ref. = 1; <30 y.); Becoming a parent × sex (ref. = women)	1.26	0.66	1.92	.06
Becoming a parent × age group (ref. = 2; 30–44 y.); Becoming a parent × sex (ref. = men)	–0.31	1.08	–0.29	.77
Becoming a parent × age group (ref. = 2; 30–44 y.); Becoming a parent × sex (ref. = women)	2.84	0.33	8.55	< .001
Becoming a parent × age group (ref. = 3; 45–54 y.); Becoming a parent × sex (ref. = men)	0.61	1.35	0.45	.65
Becoming a parent × age group (ref. = 3; 45–54 y.); Becoming a parent × sex (ref. = women)	3.76	1.18	3.18	.002
Control variables				
Sex (0 = male; 1 = female)	0.08	0.18	0.46	.64
Level of education (1–5)	0.03	0.07	0.47	.64
Previous activity duration (1–15)	–0.11	0.03	–4.07	< .001
Quotient active years	–0.49	0.52	–0.95	.34
age group (ref. = 1; <30 y.)				
age group 2 (30–44 y.)	–0.70	0.23	–3.00	.003
age group 3 (45–54 y.)	–0.71	0.23	–3.14	.002
Time (ref. = 8)				
Time 1	0.22	0.44	0.49	.62
Time 2	–0.09	0.48	–1.8	.86
Time 3	0.43	0.44	0.97	.33
Time 4	–0.58	0.54	–1.07	.29
Time 5	–0.48	0.54	–0.89	.38
Time 6	0.39	0.44	0.89	.37
Time 7	0.47	0.44	1.06	.29
Time 9	0.19	0.48	0.39	.69
Time 10	0.10	0.49	0.21	.84
Time 11	0.74	0.44	1.68	.09

	Time 12	0.50	0.45	1.12	.26
	Time 13	0.58	0.45	1.30	.19
	Time 14	0.40	0.46	0.87	.38
	Time 15	–0.11	0.51	–0.21	.83
Random effects		*Variance*	*SD*		
Person		0.30	0.55		

Note: *N* = 12689 observations, *n* = 1388 persons; SE = standard error, SD = standard deviation.

[1]To compare persons with and without experiencing a life event for a specific age group and sex, the reference category was changed to these values (Jaccard, 2001). The model and its values stays the same.

Supplementary Table S13: Multilevel discrete–time event–history analysis for **starting vocational training on terminating LTPA**. The model presented due to the best fit is calculated with the interaction effects for ending a relationship × age group, whereas the interaction with sex does not improve model fit.

Fixed effects	logit	*SE*	*z* value	*P*
Intercept	–10.09	2.17	–4.65	< .001
Starting vocational training (15–44 years)				
Starting voc. training × age group (ref. = 1; 30 y.)	0.17	0.43		.68
age group 2 (30–44 y.)	1.90	0.66		.004
changing reference category[1]				
Starting voc. training × age group (ref. = 2; 30–44 y.)	2.07	0.51	4.05	< .001
Control variables				
Sex (0 = male; 1 = female)	0.34	0.25	1.33	.18
Level of education (1–5)	0.07	0.10	0.69.49	
Previous activity duration (1–15)	–0.04	0.04	–1.08	.28
Quotient active years	–2.23	0.82	–2.73	.006
age group (ref. = 1; <30 y.)				
age group 2 (30–44 y.)	–0.59	0.27	–2.20	.03
Time (ref. = 8)				
Time 1	0.40	0.57	0.72	.47
Time 2	0.15	0.61	0.24	.81
Time 3	0.27	0.60	0.45	.65
Time 4	–0.31	0.69	–0.45	.65
Time 5	–0.92	0.85	–1.08	.28
Time 6	0.46	0.60	0.77	.44
Time 7	0.59	0.58	1.02	.31
Time 9	0.25	0.66	0.38	.70
Time 10	–0.03	0.70	–0.04	.97
Time 11	0.79	0.60	1.32	.19
Time 12	0.36	0.64	0.57	.57
Time 13	0.90	0.58	1.54	.12
Time 14	0.69	0.62	1.11	.27
Time 15	–0.01	0.70	–0.01	.99
Random effects	*Variance*	*SD*		
Person	0.87	0.93		

Note: *N* = 5385 observations, *n* = 847 persons; SE = standard error, SD = standard deviation.

[1]To compare persons with and without experiencing a life event for a specific age group the reference category was changed to this value (Jaccard, 2001). The model and its values stays the same.

Supplementary Table S14: Multilevel discrete–time event–history analysis for **starting a job on terminating LTPA**. The model presented is calculated without interaction effects for the life event with gender and/or age groups, because this does not improve model fit.

Fixed effects	logit	*SE*	*z* value	*P*
Intercept	–5.49	1.10	–4.98	< .001
Starting a job (15–70 years)	0.02	0.26		.93
Control variables				
Sex (0 = male; 1 = female)	0.29	0.15	1.87	.06
Level of education (1–5)	–0.01	0.06	–0.15	.88
Previous activity duration (1–15)	–0.11	0.02	–4.81	< .001
Quotient active years	–0.39	0.42	–0.94	.35
age group (ref. = 1; <30 y.)				
age group 2 (30–44 y.)	–0.37	0.21	–1.74	.08
age group 3 (45–59 y.)	–0.72	0.20	–3.49	< .001
age group 4 (60–70 y.)	–1.10	0.29	–3.80	< .001
Time (ref. = 8)				
Time 1	0.12	0.41	0.29	.77
Time 2	0.01	0.42	0.01	.99
Time 3	0.29	0.40	0.73	.46
Time 4	–0.53	0.49	–1.09	.27
Time 5	–0.25	0.45	–0.54	.59
Time 6	0.44	0.39	1.13	.26
Time 7	0.40	0.39	1.02	.31
Time 9	0.01	0.43	0.03	.98
Time 10	0.24	0.41	0.59	.55
Time 11	0.69	0.38	1.83	.07
Time 12	0.40	0.39	1.02	.31
Time 13	0.34	0.40	0.85	.40
Time 14	0.51	0.39	1.32	.19
Time 15	0.26	0.40	0.65	.52
Random effects	*Variance*	*SD*		
Person	0.24	0.49		

Note: *N* = 19690 observations, *n* = 1744 persons; SE = standard error, SD = standard deviation; Time 16 had to be removed from the calculation because there are no terminations there.

Supplementary Table S15: Multilevel discrete–time event–history analysis for **ending a job on terminating LTPA**. The model presented is calculated without interaction effects for the life event with gender and/or age groups, because this does not improve model fit.

Fixed effects	logit	*SE*	*z* value	*P*
Intercept	–5.59	1.12	–5.01	< .001
Ending a job (15–70 years)	0.82	0.22	3.67	< .001
Control variables				
Sex (0 = male; 1 = female)	0.27	0.15	1.76	.08
Level of education (1–5)	–0.01	0.06	–0.23	.82
Previous activity duration (1–15)	–0.11	0.02	–4.80	< .001
Quotient active years	–0.39	0.42	–0.93	.35
age group (ref. = 1; <30 y.)				
age group 2 (30–44 y.)	–0.34	0.21	–1.64	.10
age group 3 (45–59 y.)	–0.67	0.20	–3.29	< .001
age group 4 (60–70 y.)	–0.96	0.29	–3.35	< .001
Time (ref. = 8)				
Time 1	0.13	0.41	0.31	.75
Time 2	0.01	0.42	0.02	.98
Time 3	0.29	0.40	0.73	.47
Time 4	–0.52	0.49	–1.06	.29
Time 5	–0.24	0.45	–0.54	.59
Time 6	0.44	0.39	1.14	.25
Time 7	0.40	0.39	1.02	.31
Time 9	0.01	0.43	0.02	.98
Time 10	0.25	0.41	0.61	.54
Time 11	0.68	0.38	1.80	.07
Time 12	0.41	0.39	1.05	.29
Time 13	0.31	0.40	0.77	.44
Time 14	0.50	0.39	1.29	.20
Time 15	0.21	0.41	0.52	.60
Random effects	*Variance*	*SD*		
Person	0.28	0.53		

Note: *N* = 19355 observations, *n* = 1742 persons; SE = standard error, SD = standard deviation; Time 16 had to be removed from the calculation because there are no terminations there.

Supplementary Table S16: Multilevel discrete–time event–history analysis for **retirement on terminating LTPA**. The model presented is calculated without interaction effects for the life event with gender and/or age groups, because this does not improve model fit.

Fixed effects	logit	*SE*	*z* value	*P*
Intercept	–3.57	1.66	–2.15	.03
Retirement (50–72 years)	–0.36	0.74	–0.49	.62
Control variables				
Sex (0 = male; 1 = female)	0.48	0.26	1.82	.06
Level of education (1–5)	–0.12	0.10	–1.20	.23
Previous activity duration (1–15)	–0.13	0.03	3.80	< .001
Quotient active years	0.68	0.62	1.08	.28
age group (ref. = 1; 50–59 y.)				
age group 4 (60–72 y.)	–0.40	0.27	–1.45	.15
Time (ref. = 8)				
Time 1	–0.74	0.84	–0.88	.38
Time 2	–0.07	0.68	–0.10	.92
Time 3	–0.07	0.68	–0.11	.92
Time 4	–1.53	1.10	–1.40	.16
Time 5	0.01	0.64	0.01	.99
Time 6	0.34	0.59	0.58	.56
Time 7	0.01	0.64	0.01	.99
Time 9	–0.97	0.84	–1.16	.25
Time 10	0.41	0.57	0.71	.48
Time 11	0.39	0.58	0.68	.50
Time 12	–0.10	0.64	–0.16	.87
Time 13	–0.60	0.73	–0.82	.41
Time 14	0.51	0.56	0.91	.36
Time 15	0.46	0.58	0.79	.43
Random effects	*Variance*	*SD*		
Person	0.15	0.38		

Note: *N* = 10653 observations, *n* = 1222 persons; SE = standard error, SD = standard deviation; Time 16 had to be removed from the calculation because there are no terminations there.

Supplementary 17: Multilevel discrete–time event–history analysis for **simultaneously occurring life events on terminating LTPA**. The model presented due to the best fit is calculated with the interaction effects for simultaneously occurred life events × age group, whereas the interaction with sex does not improve model fit.

Fixed effects	logit	*SE*	*z* value	*P*
Intercept	–5.99	1.15	–5.22	< .001
Simul. occurring life events (15–70 years)				
Simul. occurring life events × age group (ref. = 1; <30 y.)	0.30	0.15	2.04	.04
age group 2 (30–44 y.)	0.53	0.21	2.50	.01
age group 3 (45–59 y.)	–0.13	0.28	–0.48	.63
age group 4 (60–70 y.)	0.09	0.47	0.20	.84
changing reference category[1]				
Simul. occurreing life events × age group (ref. = 2; 30–44 y.)	0.83	0.15	5.39	< .001
Simul. occurring life events × age group (ref. = 3; 45–59 y.)	0.17	0.24	0.71	.48
Simul. occurring life events × age group (ref. = 4; 60–70 y.)	0.40	0.45	0.88	.38
Control variables				
Sex (0 = male; 1 = female)	0.31	0.15	2.03	.04
Level of education (1–5)	–0.03	0.06	–0.46	.64
Previous inactivity duration (1–15)	–0.10	0.02	–4.23	< .001
Quotient active years	–0.43	0.43	–0.10	.32
age group (ref. = 1; <30 y.)				
age group 2 (30–44 y.)	–0.50	0.26	–1.89	.06
age group 3 (45–59 y.)	–0.54	0.24	–2.23	.03
age group 4 (60–70 y.)	–1.15	0.30	–3.83	< .001
Time (ref. = 8)				
Time 1	0.23	0.42	0.54	.59
Time 2	0.17	0.44	0.40	.69
Time 3	0.46	0.41	1.12	.26
Time 4	–0.35	0.50	–0.71	.48
Time 5	0.11	0.44	0.25	.80
Time 6	0.60	0.40	1.50	.13
Time 7	0.62	0.40	1.55	.12
Time 9	0.11	0.44	0.26	.80
Time 10	0.33	0.42	0.80	.43
Time 11	0.76	0.39	1.94	.05
Time 12	0.59	0.40	1.48	.14

	Time 13	0.53	0.40	1.31	.19
	Time 14	0.63	0.40	1.59	.11
	Time 15	0.39	0.41	0.95	.34
Random effects		*Variance*	*SD*		
Person		0.39	0.62		

Note: N = 21783 observations, n = 1743 persons; SE = standard error, SD = standard deviation.

[1]To compare persons with and without experiencing a life event within the age group, the reference category was changed to this age group (Jaccard, 2001). The model and its values stays the same; Time 16 had to be removed from the calculation because there are no terminations there.
